# On-Field Ski Kinematic According to Leg and Discipline in Elite Alpine Skiers

**DOI:** 10.3389/fspor.2020.00056

**Published:** 2020-05-15

**Authors:** Marine Alhammoud, Clint Hansen, Frederic Meyer, Christophe Hautier, Baptiste Morel

**Affiliations:** ^1^French Ski Federation, Annecy, France; ^2^Inter-University Laboratory of Human Movement Biology (EA 7424), University Claude Bernard Lyon 1, Lyon, France; ^3^Surgery Department, Aspetar Orthopaedic and Sports Medicine Hospital, Doha, Qatar; ^4^Department of Neurology, Christian-Albrechts-Universität zu Kiel Medizinische Fakultat, Kiel, Germany; ^5^Institute of Sport Science, University of Lausanne, Lausanne, Switzerland; ^6^Laboratory “Movement, Interactions, Performance” (EA 4334), Le Mans University, Le Mans, France; ^7^Inter-University Laboratory of Human Movement Biology (EA 7424), Savoie Mont Blanc University, Chambéry, France

**Keywords:** winter sports, angular velocity, knee angle, knee injury prevention, quasi-isometric contraction

## Abstract

This study used wireless technology to investigate joint kinematic characteristics of the four alpine skiing disciplines. Knee and hip angles were measured in 20 national team alpine skiers during 253 ski runs under FIS regulation, including: 85 Slalom (SL), 123 Giant Slalom (GS), 29 Super Giant Slalom (SG), and 16 Downhill (DH). Data were analyzed by outside (OL, *n* = 2,087) and inside leg (IL, *n* = 2,015). The proportion of concentric and eccentric phases *(extension and flexion respectively for the knee extensors)* as well as the proportion of the quasi-isometric phase defined between ±20°.s^−1^ depended on the discipline in interaction with the IL/OL (*p* < 0.001). The results showed a lower knee quasi-isometric duration on OL in SL (11%) than other disciplines (DH: 38%; SG: 42%; GS: 34%, *p* < 0.001, *d* > 1.8), suggesting a highly dynamic style. Quasi-isometric mode was significantly longer on OL than IL in GS (34 vs. 20%, *p* < 0.001, *d* = 1.16) and SG (42 vs. 28%, *p* < 0.001, *d* = 1.11) but was significantly longer on IL than OL in SL (19 vs. 11%, *p* < 0.001, *d* = 0.64). Thus, GS and SG showed similarities, with a significantly faster knee eccentric mean angular velocity on IL compared to OL (GS −58 vs. −54°.s^−1^, SG −52 vs. −45°.s^−1^, *p* < 0.001, *d* ≥ 0.22) whereas SL showed an opposite pattern (−72 vs. −89°.s^−1^, *p* < 0.001, *d* = 1.10). The quasi-isometric phase was overlooked in previous studies but is crucial to consider. The current data may be used to train the outside and inside leg specificities incorporating discipline-specific contraction modes and exercises.

## Introduction

The muscular work of the skier was historically described at slow angular velocity during knee flexion (eccentric work of the knee extensors) and extension phases (concentric work of the knee extensors) (Berg et al., 1995). Eccentric contractions resist the compressive forces while traveling down the slope and were previously reported as both longer and of higher intensity than the concentric actions during turns (Berg et al., 1995). However, equipment design and movement pattern have markedly changed during the last decades, especially with the introduction of parabolic skis (carving) (Raschner et al., 2001). These developments have brought this eccentric predominance into question as quasi-isometric and concentric components have been described during carved turns (Kröll et al., 2015a; Minetti, 2016). For example, it was suggested that the bi-articular *rectus femoris* maintained a nearly constant length in female practicing recreational ski whereas the *vastus lateralis* lengthened during the first phase of inside leg (IL) turn (eccentric work), then shortened during the edge change phase (concentric work) and contracted isometrically on the subsequent outside leg (OL) phase (Kröll et al., 2010). The evolution in the joint kinematic aspects of the ski turn with ski carving has been described in Giant Slalom (GS) (Kröll et al., 2015a) but never in the speed disciplines.

Alpine skiing includes four disciplines, with most skiers specialized in technical [Slalom (SL) and GS] or speed disciplines [Super Giant (SG) and Downhill (DH)] (Gilgien et al., 2018b). The GS often serves as a midpoint between SL and SG/DH (Turnbull et al., 2009) due to the technological limitations in recording speed disciplines. Yet, the principles of “kinematic, kinetic, and neuromuscular correspondence” and “coordinative affinity” (Kröll et al., 2015a) should be taken into consideration for sports-specific training; therefore, exercises must be similar to the movements characterizing the discipline (Müller et al., 2000). Ski-specific exercises for technical training include inline skating (Zeglinksi et al., 1998; Kröll et al., 2005), eccentric bike (Gross et al., 2010), or practice on ski ergometers (Panizzolo et al., 2013; Moon et al., 2015; Stöggl et al., 2018). However, DH skiers may require more prolonged isometric maximum- and endurance-force training whereas SL skiers have a greater need for explosiveness with higher rate of force development and dynamic style (Kröll et al., 2015b).

A “quasi-static” component to skiing was already suggested in the technical disciplines due to evidence of thigh muscles co-contraction (Hintermeister et al., 1995). Nevertheless, the quasi-isometric phase was overlooked in the old studies using a double partition of concentric vs. eccentric phases defined between minimal and maximal or maximal to minimal knee joint angles, respectively (Berg et al., 1995; Berg and Eiken, 1999). In addition, previous studies concentrated on the outside phase of experimentally controlled ski cycles (Berg et al., 1995; Hintermeister et al., 1995; Berg and Eiken, 1999) and limited kinematic information is available on the IL (Kröll et al., 2010, 2015a,b), despite the recognized importance of leg independence in alpine skiing (LeMaster, 2010). In this manuscript, we refer to the “quasi-isometric, eccentric and concentric” phases of the ski cycle at the macroscopic knee joint level which may reflect the behavior of the muscle-tendon unit for the monoarticular muscles (*vastii*), but not for the polyarticular muscles (*rectus femoris* and hamstrings) which are impacted by the hip position (Hawkins and Hull, 1990).

In summary, given the distinct nature of the eccentric/concentric/isometric contraction regimens (Higbie et al., 1996), and in the absence of joint kinematic comparison between disciplines, it appears necessary to study the contraction modes and angle-related parameters specific to leg and discipline in order to reproduce its characteristics during dryland trainings. In this context, the knee angular behavior has been suggested as one of the most important parameters for performance, albeit highly variable when measured at minimum radius of the turns in SL (Pozzo et al., 2010). Recent developments in technology including wireless sensors and miniaturized datalogger have made it possible to continuously record angular behavior during complete ski runs. Therefore, the aim of the current study was to investigate the joint kinematic characteristics of elite alpine skiers according to leg and discipline. It was hypothesized that the discipline and leg will affect the knee and hip angles, the angular velocities and the contraction mode durations. It was further hypothesized that a marked quasi-isometric component would be evidenced in the speed disciplines compared with the technical disciplines.

## Materials and Methods

### Participants

Twenty French national team alpine skiers [9 females (23 ± 2 years, 169 ± 5 cm, 64.3 ± 4.7 kg) and 11 males (24 ± 5 years, 178 ± 7 cm, 76.2 ± 7.0 kg)] participated in this study. Skiers were competing in either World Cup (*n* = 13) or Europa Cup (*n* = 7), as Technicians (*n* = 15) or Speed Specialists (*n* = 5), with an average FIS points of 12 ± 7 (the lower the better). The study was approved by the university ethics board and participants provided written informed consent prior to commencement. All procedures conformed to the standards of the Declaration of Helsinki. Participants were provided with prior medical clearance to compete.

### General Procedure

Hip and knee angles as well as center of mass acceleration were synchronously measured during 253 ski runs (85 SL, 123 GS, 29 SG, and 16 DH), whilst participants followed their regular ski training program. A total of 49 skier-sessions were obtained corresponding to the triplet “skier, day of testing and discipline.” All data were collected during 2 consecutive winter seasons (2016-2017), in the ski resort of Cerro Castor (Ushuaïa, Argentina). Athletes skied in accordance with FIS regulations including using their own equipment and every run (turn length and gate offset) was timed.

### Measurements

#### Measurement of Angles

**Figure 1:**
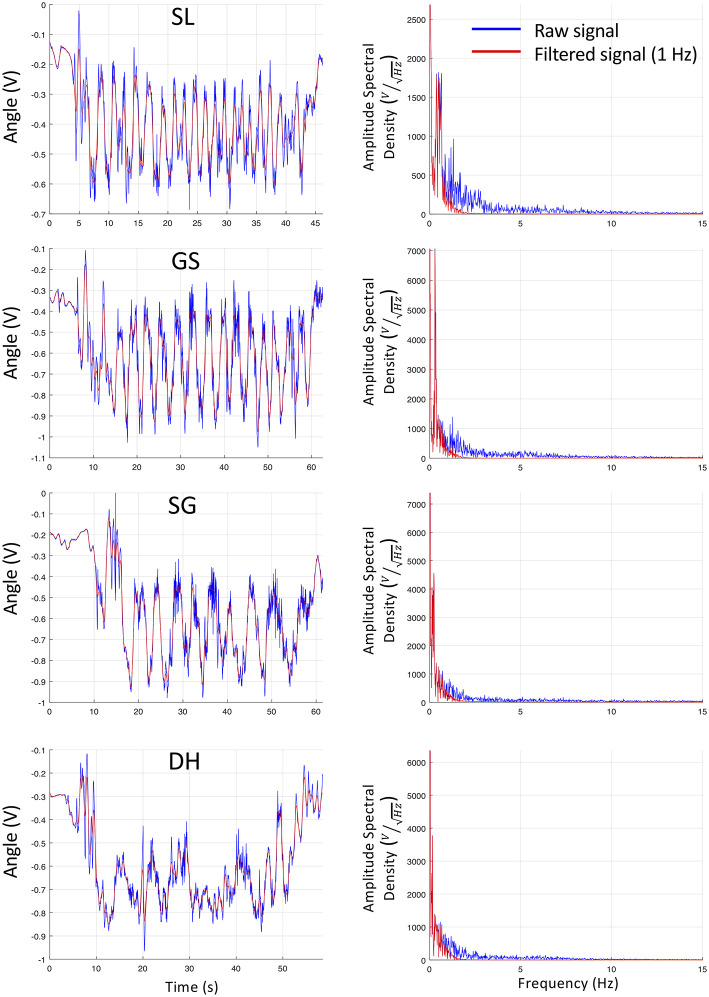
Example of raw and filtered signals in the four alpine disciplines (SL Slalom, GS Giant Slalom, SG Super Giant, DH Downhill). **(Left)** Raw (V) knee angle (blue) and filtered signal (red) in the temporal domain. **(Right)** raw knee angle (blue) and filtered signal (red) in the spectral domain (Fast Fourier Transform) of the amplitude spectral density expressed in V/$\sqrt{Hz}$.

Electrogoniometers (SG150, Biometrics, Newport, UK) were positioned on the right knee and hip. The goniometers were individually calibrated on the participants the day before the ski run using a video analysis (kinovea, www.kinovea.org) based on three positions (standing, seated, crouched) and their location was marked with an indelible marker. Data were recorded at 148.15 Hz via a portable wireless data logger (TPM, Trigno Personnal Monitor, Delsys) using a wireless Trigno Goniometer Adapter (Delsys, Boston, USA). In the absence of filtering consensus in the ski literature, a lowpass filter (4th order, Butterworth) with a cut-off frequency set at 1 Hz was retained after inspection of the data with Fast Fourier Transform and logarithmic Bode plots (Figure 1). During SL, this strong filtering induced a discrete mitigation of the angle amplitude without aliasing (Figure 1), as the 0.5 Hz spectral component was reduced by 0.5 dB only (i.e., 5% amplitude reduction). Importantly, the chosen filter was very selective (−80 dB/decade), representing a good compromise to protect the useful signal (mean natural frequencies: SL 0.54 Hz, GS 0.34 Hz, SG 0.25 Hz, DH 0.21 Hz) while removing the noise of the unwanted vibrations (2 Hz noise reduced by 24.5 dB, i.e., 94% amplitude reduction Figure 1). The phase/frequency relationship of the transfer function filter was constantly equal to zero meaning that there was no temporal distortion as no delay occurred at any frequencies.

#### Cycle Determination

The TPM was located on the skiers abdomen under the racing suit and tri-axial acceleration was recorded at 148.15 Hz, from which the resultant acceleration (AccR) was determined: $AccR\;\left(g\right)=\;\sqrt{x^{2}+y^{2}+z^{2}}$. The AccR was low-pass filtered (fourth-order Butterworth filter with a cut-off frequency of 1 Hz). The minimal (inflection point of the signal derivative) was used to automatically detect the turn switch (Supej et al., 2003; Fasel et al., 2016). This offered good turn detection in all disciplines without noise contamination by the vibrations of the material and/or the slope, as frequencies over 2 Hz have been identified as undesirable vibrations during carving (Nemec et al., 2001).

The data were time-normalized to 100% of each IL/OL. The first/last cycles and the figures (double or triple gates, banana, jumps) were discarded from the analyses to account for the push-off phase and remove the specific characteristics of the figures. Maximal acceleration was not reached after the first gate but the knee angle curves were already in the variability range of the following cycles after two turns (one cycle). There were a few occurrences of goniometer's signal issues (e.g., broken after pole contact) and the corresponding data were systematically discarded. A total of 2087 OL/2015 IL were analyzed for the knee including 686/639 SL, 1113/1157 GS, 221/166 SG, and 67/53 DH. A total of 1599 OL/1581 IL were analyzed for the hip including 482/465 SL, 948/988 GS, 151/104 SG, and 18/24 DH.

### Data Analysis

The minimum and maximum angles were computed on the IL and OL for the right knee and hip. As skiing involves resisting gravity and compression, the movement of flexion was assumed to occur in eccentric mode and extension in concentric mode for the knee extensors (Berg et al., 1995; Federolf et al., 2009; Kröll et al., 2015a). Indeed, due to centripetal forces the skiers muscles are loaded eccentrically and the control of the kinetic energy occurs via eccentric muscle activity (Vogt and Hoppeler, 2014). This seems to be an inherent principle of alpine skiing (Vogt and Hoppeler, 2014). However, during knee flexion, a relaxation of the quadriceps without eccentric activity could theoretically occur. Interpretation of the contraction regimen without EMG activity must therefore be done considering this simplification is often observed in the ski literature (Berg et al., 1995; Federolf et al., 2009; Panizzolo et al., 2013; Kröll et al., 2015b).

The first derivative of the knee angle was used to compute the angular velocity and to define the contraction regimen phases. Angular velocities between ±20°.s^−1^ were defined as quasi-isometric, containing both slow eccentric and concentric motions. Angular velocities below and above this window (range = 40°.s^−1^) were defined as eccentric and concentric, respectively. In other words, a knee flexion contains a slow eccentric motion and a knee extension contains a slow concentric motion both classified as quasi-isometric contraction. Lastly, the minimum and maximum angles were also used to describe the flexion and extension phases (Table 2) to allow comparison with previous literature (Berg et al., 1995; Nilsson and Haugen, 2004; Panizzolo et al., 2013).

**Figure 2:**
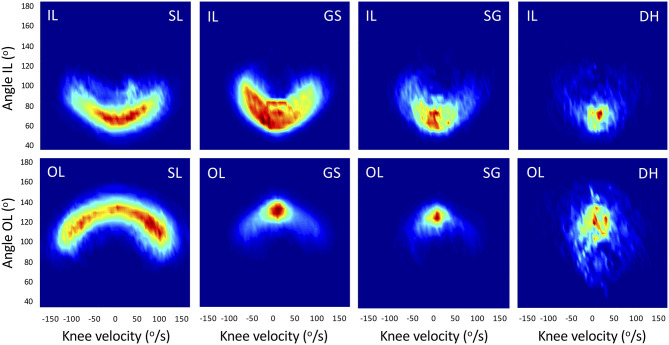
2D distribution of the knee angular position (y-axis) and velocity (x-axis) for Slalom (SL), Giant Slalom (GS), Super Giant (SG), and Downhill (DH). This representation shows at which angular position a given angular velocity mainly occurred. Deep blue is 0 (no occurrence), dark red is maximum occurrence within the discipline and leg (linear color scale, arbitrary units). IL, inside leg; OL, outside leg.

The absolute (in ms) and relative durations (in % of cycle duration) spent in eccentric (flexion, angular velocity < -20°.s^−1^), concentric (extension, angular velocity >20°.s^−1^), or quasi-isometric mode (angular velocity between −20 and +20°.s^−1^) were analyzed. The maximal and mean eccentric and concentric angular velocities were then computed. Two-dimensional density plots were realized for IL and OL in each discipline. They were created using bivariate histograms to examine at which angular position a given angular velocity mainly occurred. A linear color scale intensity was used to show maximum occurrence normalized per turn phase within each discipline (Figure 2).

### Statistics

Due to the repeated observations, the measurement independence condition is not fulfilled and a classical analysis of variance cannot be applied. Consequently, a mixed linear model was fitted with a random effect on the skier-session (ID) factor. Fixed effects were analyzed for discipline (DH, SG, GS, and SL) and leg (IL, OL) along with the interaction between these two effects using the following model: Y ~ Discipline + IL_OL + Discipline x IL_OL + (1|ID). For the maximum and minimum angles, the values were extracted from the OL and IL, respectively and analyzed as Y ~ Discipline + (1|ID). This method accounts for data autocorrelation due to the repetition of runs, the intermittent missing data after database cleaning, the unbalanced design between disciplines, and the unequal numbers of IL/OL (Cnaan et al., 1997). Data were log-transformed in case of non-normal distribution (Manning and Mullahy, 2001). Significance of both the simple and interaction fixed effects was evaluated with an analysis of variance (Restricted Maximum Likelihood - REML - estimation). After fitting the linear mixed-effect model, *post-hoc* comparisons were performed among groups with Tukey multiplicity correction. All statistical comparisons were coded in R (R Foundation for Statistical Computing, Vienna, Austria) using the *lmerTest* package (Kuznetsova et al., 2017). Estimated marginal means and *post-hoc* tests were calculated with *emmeans* package (Lenth, 2019). Effect sizes for the *post-hoc* pairwise comparison were calculated as Cohen's *d* for mixed linear model and evaluated as small (≥0.20), medium (≥0.50), and large (≥0.80) (Westfall et al., 2014; Brysbaert and Stevens, 2018). Data were expressed in mean ± standard deviation. Significance was set at *p* < 0.05 and trends were discussed when *p* < 0.10.

## Results

### Angles and Mean Angular Velocity

**Figure 3:**
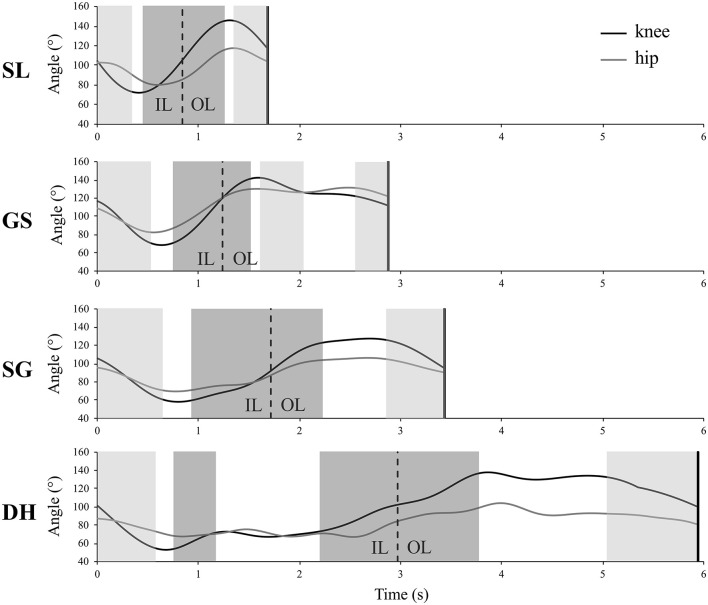
Example of 1 cycle (inside leg, IL and outside leg, OL) for 1 skier-session per discipline expressed in absolute time (s). SL, Slalom; GS, Giant Slalom; SG, Super Giant; DH, Downhill. The gray-shaded areas represent the eccentric (light gray), concentric (dark gray), and quasi-isometric (white background) phases of the knee.

An example of each alpine skiing disciplines is graphically represented along with the contraction regimen (Figure 3).

The effect of discipline was not significant for the knee max angle on OL (*p* = 0.070) or the min angle on IL (*p* = 0.161) (Table 1). The hip max angle on OL depended on discipline (*p* = 0.014) with a trend for lower values in DH and SG than GS and SL (*p* < 0.090). The min hip angle on IL did not depend on discipline (*p* = 0.117) (Table 1).

**Table 1:** Angles related parameters according to Leg and Discipline including a quasi-isometric phase between ±20°s^−1^.

	**Joint**			**Inside**	**Leg**			**Outside**	**Leg**	
			**DH**	**SG**	**GS**	**SL**	**DH**	**SG**	**GS**	**SL**
Angle (°)	Knee	Min IL/Max OL	58 ± 9^a^	60 ± 8^a^	64 ± 9^a^	67 ± 12^a^	128 ± 17^a^	127 ± 8^a^	132 ± 9^a^	129 ± 11^a^
	Hip	Min IL/Max OL	66 ± 3^a^	70 ± 6^a^	90 ± 18^a^	93 ± 21^a^	89 ± 6^a^	106 ± 9^a^	128 ± 18^a^	126 ± 15^a^
Mean angular velocity	Knee	Concentric	50 ± 19^a^	59 ± 16^ab^	65 ± 16^b^	63 ± 19^ab^	52 ± 20^a^	57 ± 14^a^	65 ± 19^a^	86 ± 22^*b^
		Eccentric	52 ± 18(^*a^)	52 ± 15^*a^	58 ± 15^*a^	72 ± 15^b^	46 ± 17^ab^	45 ± 12^a^	54 ± 16^b^	89 ± 21^*c^
	Hip	Concentric	28 ± 5^a^	36 ± 11^a^	43 ± 14^*a^	48 ± 21^a^	26 ± 4^ab^	40 ± 9^*ab^	41 ± 14^a^	55 ± 19^*b^
		Eccentric	27 ± 4^a^	33 ± 8^a^	39 ± 12^*a^	38 ± 14^a^	26 ± 4^a^	31 ± 7^a^	34 ± 10^a^	42 ± 14^*a^
Absolute duration phase (ms)	Knee	Concentric	793 ± 342^*d^	633 ± 239^*c^	553 ± 147^*b^	375 ± 123^a^	748 ± 368^c^	526 ± 195^b^	458 ± 148^a^	426 ± 81^*a^
		Quasi-iso	1042 ± 646^d^	588 ± 380^c^	298 ± 219^b^	175 ± 111^*a^	919 ± 534^c^	858 ± 354^*c^	506 ± 263^*b^	99 ± 62^a^
		Eccentric	791 ± 416^c^	776 ± 213^*c^	625 ± 173^*b^	372 ± 126^a^	726 ± 309^c^	646 ± 272^c^	494 ± 206^b^	367 ± 89^a^
		Turn duration	2626 ± 643^*d^	1998 ± 390^c^	1477 ± 255^b^	922 ± 144^*a^	2394 ± 548^d^	2030 ± 387^c^	1459 ± 216^b^	892 ± 134^a^
	Hip	Concentric	338 ± 293^ab^	402 ± 191^b^	459 ± 191^*b^	248 ± 119^a^	349 ± 299^ab^	414 ± 200^b^	301 ± 181^a^	315 ± 107^*a^
		Quasi-iso	2407 ± 584^*d^	1113 ± 446^c^	552 ± 364^b^	355 ± 218^*a^	2078 ± 423^d^	1254 ± 392^*c^	803 ± 319^*b^	249 ± 172^a^
		Eccentric	485 ± 309^*ab^	500 ± 229^*b^	468 ± 218^*b^	333 ± 197^a^	169 ± 199^a^	414 ± 246^a^	339 ± 214^a^	345 ± 151^*a^
		Turn duration	3230 ± 469^d^	2015 ± 352^c^	1480 ± 249^*b^	936 ± 156^*a^	2597 ± 588^*d^	2083 ± 38*2^c^	1443 ± 200^b^	909 ± 135^a^
Relative duration phase (% cycle)	Knee	Concentric	32 ± 15^a^	32 ± 11^*a^	37 ± 9^*b^	41 ± 13^c^	31 ± 14^b^	26 ± 9^a^	32 ± 11^b^	48 ± 7^*c^
		Quasi-iso	38 ± 21^b^	28 ± 15^b^	20 ± 13^a^	19 ± 11^*a^	38 ± 18^bc^	42 ± 15^*c^	34 ± 16^*b^	11 ± 5^a^
		Eccentric	30 ± 14^a^	40 ± 11^*b^	43 ± 11^*b^	40 ± 12^b^	31 ± 14^a^	32 ± 13^a^	34 ± 14^a^	41 ± 8^b^
	Hip	Concentric	10 ± 8^a^	20 ± 11^ab^	31 ± 12^*c^	26 ± 12^b^	12 ± 9^a^	20 ± 9^a^	21 ± 12^a^	35 ± 11^*b^
		Quasi-iso	74 ± 12^c^	54 ± 16^bc^	37 ± 22^a^	39 ± 24^*ab^	81 ± 13^b^	60 ± 15^*b^	55 ± 20^*b^	27 ± 17^a^
		Eccentric	16 ± 10^*a^	25 ± 12^*a^	32 ± 15^*a^	35 ± 20^a^	6 ± 7^a^	20 ± 12^a^	24 ± 15^a^	38 ± 16^*b^

SL, Slalom; GS, Giant Slalom; SG, Super Giant; DH, Downhill. Quasi-iso: quasi-isometric phase. Superscript letters represent post-hoc pairwise comparisons between disciplines with two disciplines with the same letters being not statistically different and a<b<c<d (mean increasing order) at p < 0.05. ^*^Inside Leg different from Outside Leg in a given discipline, while indicating the higher value. (^*^) indicates a trend with p < 0.10. Nb: Turn duration is different for Knee and Hip due to a different sample size (see Methods for details).

**Figure 4:**
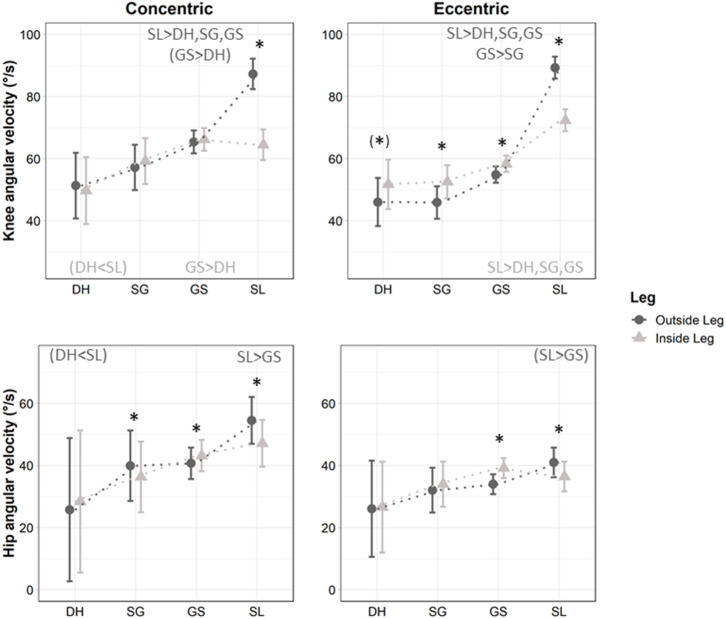
Mean concentric and eccentric angular velocity (±20°.s^−1^) for the right knee (KR) and hip (HR) during Downhill (DH), Super Giant (SG), Giant Slalom (GS), and Slalom (SL) when separated into concentric, eccentric, and quasi-isometric modes. ^*^denotes a significant difference between inside (light gray and triangles) and outside leg (dark gray and plain circles). < or > differences between disciplines for the inside (light gray) or the outside (dark gray) leg. *p* < 0.05, ( ) indicates a trend with *p* < 0.10.

The mean knee and hip angular velocities beyond ±20°.s^−1^ showed an interaction between discipline and leg (*p* < 0.001) (Table 1). *Post-hoc* analyses showed that the knee concentric velocity was higher in SL than other disciplines on OL (*p* < 0.001, large effects *d* > 1.27), but not IL (*p* > 0.638, *d* ≤ 0.25; except a trend for SL vs. DH *p* = 0.073, medium effect *d* = 0.79). The knee concentric velocity was faster in GS than DH on IL (*p* = 0.027, large effect *d* = 0.93), with only a trend on OL (*p* = 0.070, medium effect *d* = 0.75, Figure 4, Table 1). The hip concentric velocity on OL was higher in SL than GS (*p* = 0.020, large effect *d* = 1.12) and tended to be higher in SL than DH (*p* = 0.094, large effect *d* = 2.34), without differences on IL (*p* > 0.382, *d* ≤ 1.56, Figure 4, Table 1). In addition, the concentric angular velocity was faster on OL than IL for the knee in SL (*p* < 0.001, large effect *d* = 1.36), and for the hip in SL and SG (*p* ≤ 0.025, small to medium effects *d* ≥ 0.28); but was faster on IL than OL for the hip in GS (*p* < 0.001, trivial effect *d* = 0.19); without other differences (*p* > 0.231, *d* ≤ 0.21, Figure 4).

The knee eccentric velocity was significantly higher in SL than other disciplines on both legs (*p* < 0.001, large effects *d* > 0.90, Figure 4), and was also higher in GS than SG on OL (*p* = 0.018, medium effect *d* = 0.57) but not IL (*p* = 0.218, small effect *d* = 0.35). The hip eccentric velocity tended to be higher in SL than GS on OL (*p* = 0.084, medium effect *d* = 0.78) but not IL (*p* = 0.775, trivial effect *d* = 0.19). In addition, the eccentric angular velocity was faster on OL than IL for both the knee and hip in SL (*p* < 0.001, large effect knee *d* = 1.10, small effect hip *d* = 0.41); but were faster on IL than OL for the knee in GS, SG, and DH, and for the hip in GS (all *p* < 0.001, small to medium effects 0.22 ≤ *d* ≤ 0.56; except knee in DH *p* = 0.051, small effect *d* = 0.34, Figure 4).

The above variables were also calculated without accounting for the quasi-isometric phase (i.e., concentric and eccentric phases based on minimal and maximal knee angles according to Berg et al., 1995) and are presented in Table 2.

**Table 2:** Angles related parameters according to Leg and Discipline analyzed with Berg et al. (1995) cycle partition in eccentric/concentric phases.

				**Inside**	**Leg**			**Outside**	**Leg**	
			**DH**	**SG**	**GS**	**SL**	**DH**	**SG**	**GS**	**SL**
Max angular velocity (°.s^−1^)	Knee	Concentric	80 ± 40^a^	94 ± 25^a^	98 ± 26^a^	98 ± 30^a^	85 ± 37^a^	96 ± 25^a^	97 ± 30^a^	116 ± 32^*b^
		Eccentric	75 ± 33^a^	74 ± 23^*a^	82 ± 22^*a^	117 ± 27^b^	69 ± 32^a^	69 ± 22^a^	79 ± 25^a^	124 ± 30^*b^
	Hip	Concentric	32 ± 12^a^	50 ± 18^a^	58 ± 24^*a^	66 ± 33^a^	28 ± 9^a^	55 ± 17^(*a)^	54 ± 24^a^	72 ± 31^*a^
		Eccentric	29 ± 9^a^	42 ± 14^(*a)^	49 ± 21^*a^	47 ± 23^a^	21 ± 9^a^	37 ± 14^a^	40 ± 18^a^	50 ± 21^*a^
Mean angular velocity (°.s^−1^)	Knee	Concentric	36 ± 18^a^	45 ± 16^*ab^	55 ± 17^*c^	54 ± 19^bc^	39 ± 19^ab^	36 ± 11^a^	48 ± 19^b^	79 ± 22^*c^
		Eccentric	37 ± 16^a^	42 ± 15^*a^	49 ± 16^*b^	61 ± 16^c^	33 ± 16^ab^	32 ± 12^a^	40 ± 16^b^	80 ± 22^*c^
	Hip	Concentric	13 ± 4^a^	21 ± 8^a^	33 ± 15^a^	37 ± 22^a^	14 ± 4^a^	22 ± 7^a^	25 ± 13^a^	45 ± 19^b^
		Eccentric	14 ± 5^a^	22 ± 8^*a^	30 ± 14^*a^	28 ± 15^a^	11 ± 4^a^	18 ± 7^a^	21 ± 10^a^	33 ± 15^*b^
Absolute duration phase (ms)	Knee	Concentric	1321 ± 399^*d^	905 ± 284^c^	697 ± 178^b^	465 ± 125^a^	1183 ± 453^d^	943 ± 277^*c^	700 ± 224^b^	476 ± 92^a^
		Eccentric	1304 ± 493^(*d)^	1093 ± 288^c^	779 ± 182^*b^	457 ± 140^*a^	1210 ± 381^d^	1087 ± 320^c^	758 ± 250^b^	416 ± 93^a^
	Hip	Concentric	1539 ± 485^*d^	939 ± 278^c^	724 ± 202^*b^	378 ± 133^a^	1341 ± 302^d^	1015 ± 278^*c^	676 ± 265^b^	413 ± 118^*a^
		Eccentric	1690 ± 245^*d^	1076 ± 292^c^	755 ± 180^b^	558 ± 156^*a^	1256 ± 422^d^	1067 ± 373^c^	767 ± 258^b^	495 ± 141^a^
Relative duration phase (% cycle)	Knee	Concentric	51 ± 12^a^	45 ± 11^a^	47 ± 8^a^	51 ± 12^a^	49 ± 14^ab^	47 ± 12^a^	48 ± 14^*a^	53 ± 7^*b^
		Eccentric	49 ± 12^ab^	55 ± 11^b^	53 ± 8^*ab^	49 ± 12^*a^	51 ± 14^ab^	53 ± 12^b^	52 ± 14^b^	47 ± 7^a^
	Hip	Concentric	47 ± 10^ab^	47 ± 12^b^	49 ± 10^*b^	41 ± 14^a^	52 ± 8^a^	49 ± 13^a^	47 ± 17^a^	46 ± 12^*a^
		Eccentric	53 ± 10^ab^	53 ± 12^a^	51 ± 10^a^	59 ± 14^*b^	48 ± 8^a^	51 ± 13^a^	53 ± 17^*a^	54 ± 12^a^

SL, Slalom; GS, Giant Slalom; SG, Super Giant; DH, Downhill. Superscript letters represent post-hoc pairwise comparisons between disciplines with two disciplines with the same letters being not statistically different and a<b<c<d (mean increasing order) at p < 0.05. ^*^Inside Leg different from Outside Leg in a given discipline, while indicating the higher value. (^*^) indicates a trend with p < 0.10.

### Absolute Duration of the Concentric, Eccentric, and Quasi-Isometric Phases

**Figure 5:**
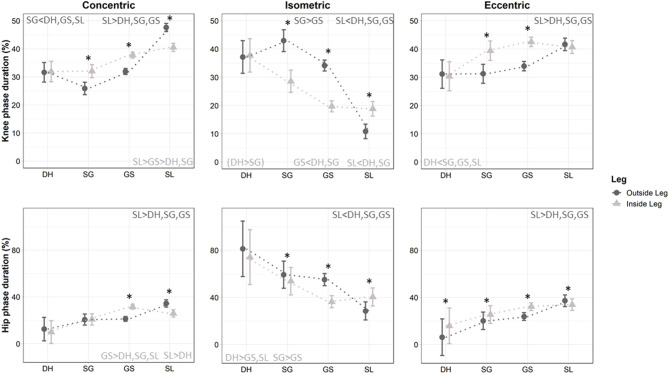
Relative duration of the eccentric, quasi-isometric and concentric phases for the knee and hip during Downhill (DH), Super Giant (SG), Giant Slalom (GS), and Slalom (SL) for the outside and inside leg. ^*^ denotes a significant difference between inside (light gray and triangles) and outside leg (dark gray and plain circles). < or > differences between disciplines for the inside (light gray) or the outside (dark gray) leg. *p* < 0.05, ( ) indicates a trend with *p* < 0.10.

The absolute duration of the concentric, quasi-isometric, and eccentric phases showed an interaction between leg and discipline (*p* < 0.001). Both legs followed a similar pattern with numerous shorter absolute durations in SL than GS, in GS than SG, and in SG than DH, but with a few minor differences between legs (Figure 5, Table 1). The concentric mode lasted longer on OL than IL for the knee and hip in SL (small effects *d* ≤ 0.41), but lasted longer on IL than OL for the knee in GS and SG (medium effects 0.64 ≤ *d* ≤ 0.72), and for the hip in GS (large effect *d* = 0.99) (all *p* < 0.001). The quasi-isometric mode lasted longer on OL than IL for both the knee and hip in GS and SG (all large effects *d* ≥ 0.90, except hip in SG medium effect *d* = 0.51), but lasted longer on IL than OL for both the knee and hip in SL (small effects 0.33 ≤ *d* ≤ 0.38) and DH (medium to large effects 0.53 ≤ *d* ≤ 1.17) (*p* < 0.006). The eccentric mode consistently lasted longer on IL than OL for the knee in GS and SG (medium effects 0.69 ≤ *d* ≤ 0.75), and for the hip in GS (medium effect *d* = 0.68), SG (small effect *d* = 0.45), and DH (large effect *d* = 1.67) (Figure 5, *p* < 0.001).

### Relative Duration of the Concentric, Eccentric, and Quasi-Isometric Phases

The relative concentric, quasi-isometric and eccentric durations in percent of the turn showed an interaction between leg and discipline (*p* < 0.001, Table 1). The relative proportion of knee concentric activity was higher in SL than GS and in GS than SG on both OL (medium to large effects 0.59 ≤ *d* ≤ 1.61) and IL (small to medium effects 0.32 ≤ *d* ≤ 0.56) (*p* < 0.019), but was equivalent in DH than SG (and thus lower to GS, *p* = 0.015, medium effect *d* = 0.60) on IL and equivalent in DH than GS (and thus higher than SG, *p* = 0.035 medium effect *d* = 0.52) on OL. The relative proportion of hip concentric activity was higher in SL than other disciplines on OL (*p* < 0.001, large effects *d* > 1.24), but was higher in GS than other disciplines on IL (*p* < 0.021, small effects vs. SL *d* = 0.45, large effects vs. SG and DH *d* > 0.97) (Figure 5, Table 1). In addition, the percentage of concentric time was higher on IL than OL for the knee in SG and GS (medium effects 0.58 ≤ *d* ≤ 0.61), and for the hip in GS (large effect *d* = 0.94); but was higher on OL than IL for the knee and hip in SL (*p* < 0.017, medium effects 0.71 ≤ *d* ≤ 0.75, Figure 5).

The relative proportion of knee quasi-isometric activity was lower in SL than other disciplines (large effects *d* > 1.86) and lower in GS than SG (medium effect *d* = 0.63) on OL (*p* < 0.001), and was lower in SL and GS (trivial effect *d* = 0.06, *p* < 0.001) and in SG and DH (*p* = 0.059, large effect *d* = 0.80) on the IL. The relative proportion of hip quasi-isometric activity was lower in SL than other disciplines on OL (*p* < 0.001, large effects *d* > 1.61); but was lower in GS than DH and SG, plus lower in SL than DH on IL (*p* < 0.040, large effects *d* > 1.00) (Figure 5, Table 1). The percentage of quasi-isometric time was higher on IL than OL for the knee and hip in SL (medium effects 0.64 < *d* < 0.65), but was higher on OL than IL for both the knee and hip in GS and SG (all large effects *d* ≥ 1.08, except hip in SG small effect *d* = 0.33) (*p* < 0.014, Figure 5).

The relative proportion of knee eccentric activity was higher in SL than other disciplines on OL (*p* < 0.002, medium to large effects *d* > 0.64), and lower in DH than other disciplines on IL (*p* < 0.024, large effects *d* ≥ 0.87) (Figure 5, Table 1). The relative proportion of hip eccentric activity was higher in SL than other disciplines on OL (*p* < 0.002, large effects *d* ≥ 1.02), without statistical differences between disciplines on IL (*p* > 0.164, despite large differences between DH vs. GS and SL *d* ≥ 1.15) (Figure 5, Table 1). The percentage of eccentric time was higher on IL than OL for the knee in GS and SG (medium effects 0.70 ≤ *d* ≤ 0.78), and for the hip in GS, SG, and DH (small to medium effects 0.39 ≤ *d* ≤ 0.69); but was higher on OL than IL for the hip in SL (*p* < 0.028, small effect *d* = 0.21) (Figure 5).

## Discussion

This study is novel and aimed to examine the knee and hip kinematics in ecologically valid skiing conditions by measuring these parameters with a portative wireless technology during the four Olympic alpine ski disciplines. In accordance with our hypothesis, the current results displayed statistical differences in joint kinematic characteristics according discipline and leg in elite skiers. Specifically, our results showed the importance of the quasi-isometric contraction mode on OL for both the knee (38, 42, 34, 11% in DH, SG, GS, and SL, respectively) and the hip (81, 60, 55, 27% in DH, SG, GS, and SL, respectively) although it was not traditionally taken into account in previous alpine skiing kinematic analysis. All disciplines with the exception of DH showed an asymmetrical behavior of the knee contraction phase durations according to their inside or outside status during the ski turn (*p* < 0.05, Figure 5). Contrary to the traditional classification into technical (SL and GS) and speed disciplines (SG and GH) and to our secondary hypothesis, the present results showed that GS and SG present similarities in their articular kinematic pattern (Figure 5) which was significantly different to both SL and DH.

### Importance of the Quasi-Isometric Phase

We observed greater knee amplitude (64–132°) in GS compared with a pioneer study (66–114°) (Berg et al., 1995) due to greater knee extension on OL (+18° of knee extension). This is likely due to a greater edge set and body inclination in the turn with the modern technique of carving with shaped skis. Knee joint angles have been related to race performance in SL with larger knee angles in the outside posture for the best skiers (Pozzo et al., 2010). Indeed, it was suggested that having these knee angular extensions at the minimum radius can account for a better modulation of the force demands derived from the external forces during the turn (Pozzo et al., 2010). The IL showed more similarities with pioneer studies as the knee angle reached a minimum of 64° (vs. 66° in Berg et al., 1995).

Hip maximum angles tended to be lower in speed than technical disciplines (*p* < 0.090) suggesting the maintenance of a tuck position without trunk extension phases. Indeed, postures that minimize the exposed frontal area of a skier are a key factor to reduce aerodynamic drag, thereby elevating velocity and reducing overall run time (Supej and Holmberg, 2019). Aerodynamic drag becomes more important as the speed increases (e.g., from SL to DH) (Gilgien et al., 2018a), whereas ski-snow friction is relatively more important at slower speeds, particularly when turning, as the ski-snow friction dissipates most of the energy during SL and GS (Supej et al., 2013). However, guiding the skis smoothly remains the priority in the speed events (Supej and Holmberg, 2019). The current results suggest that this smooth steering is executed in the aerodynamic tuck position as characterized by small hip angles (Table 1).

However, more than the extreme angular positions which were rather similar between the disciplines, the angular velocities, and/or duration of the contraction modes may be more discriminant. Indeed, the functional phase durations (IL/OL) were increasingly shorter from DH to SG, to GS and then to SL leading to faster muscle contractions for both the eccentric and concentric modes (Table 1). A kinetic study has previously shown that this shorter duration in SL did not affect the maximum ground reaction force (~3.15 body weight) but decreased the mean total ground reaction force as compared to GS (Kröll et al., 2015b). Moreover, the shorter SL turns decreased the time available for independent leg action, leading to a more balanced distribution between the two legs with synchronous legs loading in SL (Kröll et al., 2015b).

In order to compare the present results to those of Berg and Eiken (1999), we extracted angle data from the figures using Web Plot Digitizer (Rohatgi, 2015) and computed the angular velocities. These reconstructed angular velocity values (mean concentric/eccentric knee angular velocities of 32/-28°.s^−1^, 46/-34°.s^−1^, and 56/-57°.s^−1^ in SG, GS, and SL, respectively) were in accordance with an increasing velocity from SG to SL observed in the present study. However, a higher mean eccentric contraction velocity was observed on OL, mainly for the technical disciplines in the present study compared to a traditional analysis of our data (Table 2). Considerable changes in ski racing during the past decades (carving skis, skier's velocity) probably account for this angular velocity increase. The reconstructed (see above, Rohatgi, 2015) angular velocity curve from Kröll et al. (2015a,b) suggest a mean angular velocity across the whole ski cycle for concentric/eccentric modes close to the present data [47/-44°.s^−1^ (Kröll et al., 2015a) and 42/-47°.s^−1^ (Kröll et al., 2015b)] (Table 2). However, by averaging the angular velocity in the same manner as traditional ski research, quasi-isometric contractions were not taken into consideration. This could lead to (i) overlooking a key component of the speed disciplines; and (ii) underestimation of the mean velocity during dynamic contractions (Table 1). As such, we proposed to separate the angular velocities with a threshold of ±20°.s^−1^ to identify a quasi-isometric phase. Such characterization showed a quasi-isometric component that significantly depended on discipline with an interaction of the leg (Figure 5). For example, SL had a significantly lower amount of time spent in quasi-isometric than all other disciplines on OL knee and hip, suggesting a highly dynamic style.

Nevertheless, one should keep in mind that these velocities are rather slow compared to values previously reported in running (Struzik et al., 2016) or team sports (Ball, 2008) with knee angular velocity reaching ~1,000°.s^−1^ to ~1,500°.s^−1^ respectively (Figure 4). This difference can be attributed to the fact that both feet are in contact with the ground in alpine skiing whereas most sports alternate right/left ground contact with (e.g., running) or without (e.g., walking) a suspension phase. As such, alpine skiing doesn't theoretically allow for open chain movements except during jumps or micro-movements due to vibrations. Alpine skiing is thus characterized by slow dynamic motions at high loads even on IL (Meyer et al., 2019) rather than pure isometric efforts. Albeit one could argue that those velocities remain relatively close to the quasi-isometric mode as compared to other sports, it is important to acknowledge than even small differences in velocities have important neuromuscular consequences. For example, two slow angular velocities (30 and 90°.s^−1^) led to different fatigue characteristics (Morel et al., 2019). As such, dry-land training should match the angular velocities and contraction times reported in the current study per discipline.

### Inside and Outside Leg Specificities Among Alpine Skiing Disciplines

Racers have always known that the best functional technique in skiing is based on using their legs independently (LeMaster, 2010). The current data reinforce the need to analyze each leg separately. In the current study, mean knee angular velocities for the knee were higher with large effects on OL than IL in SL both eccentrically and concentrically. However, the reverse pattern was seen in GS and SG with higher mean angular eccentric velocities (small effects) for the knee on IL than OL (Table 1). Thus, all disciplines except for DH showed an asymmetrical behavior of the legs according to their inside or outside status during the ski turn. Using pressure insoles, it has previously been reported a load of ~2 body weight on OL and ~0.75 body weight on IL in GS (Kröll et al., 2015b). This suggests that the load on IL remains substantial, especially considering the flexed knee angles. Moreover, the extended OL may benefit from more skeletal support, while the flexed IL requires more muscular support (Hintermeister et al., 1997), and the muscle activity required to stabilize the flexed leg may therefore approach that of the extended leg, even if the latter is supporting greater force in GS (Falda-Buscaiot et al., 2017; Meyer et al., 2019). Taken together, these results suggest the importance of training at the closed knee angles observed on IL (Table 1, Figure 5). Both legs work independently during the turn with a more extended OL to resist the pressure and an IL more bent due to inclination. During the subsequent transition phase, both legs reached the same position. Thus, the outside knee was more extended during the turning phase than at the turn switch where the skis slide flat without pressure.

Mean hip angular velocities were higher on OL than IL in SL both eccentrically (small effect) and concentrically (medium effect). A reverse pattern was also seen in GS with higher values on IL than OL. No asymmetry was observed in DH and SG, except concentrically in SG where a higher mean angular velocity was seen on OL than IL (small effect). Thus, the hip displayed an asymmetrical pattern in the technical disciplines, almost no asymmetry in SG and a marked symmetry in DH (Figure 4).

In SG and GS, more relative time was spent in concentric and eccentric modes for the knee on IL than OL (medium effects), meaning that in the open angles, less time was spent in concentric and eccentric modes to the benefit of quasi-isometric mode (Figure 5). The IL (closed knee angles) showed a more dynamic style than OL (open angles) which displayed a more marked quasi-isometric component. Contrary to DH and SL, quasi-isometric mode was markedly prevalent in GS and SG on OL (GS 34% and SG 42%, Figure 5). Moreover, this marked quasi-isometric OL seems to be also isotonic as suggested by the observations from Meyer et al. (2019) showing that maximal vertical forces (~12 N.kg^−1^) were attained at 30% of the turn and were thereafter maintained until 70% of the cycle in GS. The opposite pattern was seen in SL as quasi-isometric mode was more marked on IL compared with OL (medium effects), albeit occupying only 19% of relative time on IL vs. ~40% in eccentric and concentric modes. The DH showed a balanced pattern without any bilateral difference, and a predominance of quasi-isometric mode at the knee (38% on both IL and OL) and hip joint (74% on IL, 81% on OL). The hip works more eccentrically on IL than OL in DH/SG/GS (small to medium effects), while SL displayed a reverse pattern (small effect). In SL, we could speculate that the OL must come back quicker than the IL to start the new turn. Albeit the practical significance of the asymmetries of knee angular behavior in ski training remains to be elucidated, the present data propose new viewpoints on the outside and inside leg specificities.

### Questioning of the Traditional Technical/Speed Disciplines Classification

Interestingly, the traditional partition between technical and speed disciplines may be questioned as GS and SG presented similarities (Figure 2), showing both a significantly faster knee eccentric mean angular velocity on IL than OL (small effects) whereas SL showed an opposite pattern (large effect). They also showed a longer quasi-isometric phase on OL than IL (large effects, Figure 5). This suggests that polyvalent skiers practicing GS and SG may develop a common and even opposite pattern to Slalomers. Thus, a new group pooling SG and GS emerged from the present analysis. DH showed symmetrical technical features (mean angular velocities, time spent in quasi-isometric/eccentric/concentric modes) whereas SG/GS were clearly asymmetrical. SL was also asymmetrical with the inverse pattern than SG/GS group. We could speculate that the “polyvalent” skiers practicing GS and SG may present some similarities in their muscular properties. These results challenged the traditional partition of the speed (DH/SG) and technical (SL/GS) disciplines, giving a rationale for the polyvalent group including GS and SG that was previously created at the French Ski Federation. Whether the inside and outside leg specificities are related to the performance of the athletes remains to be elucidated as well as the facility to switch from a profile to another.

### Interest of 2D Density Plots Visualization and Synthetic Overview

The 2D density plots (Figure 2) are proposed as an original qualitative method for data visualization to illustrate the statistical results discussed above that may be used by physicians and coaches. For example, we could see that SL displayed a predominance of slow concentric and quasi-isometric speeds on IL but a more dynamic distribution on OL with prevalent concentric and eccentric speeds (at ±~150°.s^−1^). SL seems to be the most complete discipline because of the broader range of angular velocities and knee angles in use whereas on the opposite side DH required extremely low speed/quasi-isometric mode on both legs. As discussed above, SG and GS showed similarities. In GS, the time spent in quasi-isometric mode on OL (~130°) was highly prevalent. In SG, there was two prevalent zones corresponding to the quasi-isometric mode both on OL (~120°) and IL (~60°). There is an inherent variability in alpine skiing in the knee angular position (Pozzo et al., 2010), higher than variability seen in cycling or running for joint angles, cycle-to-cycle duration, and stride time (Padulo et al., 2012; Connick and Li, 2015). The 2D plot representation may be generalized to other sports to summarize various characteristic patterns and their variability.

### Methodological Considerations

For the practical applications, it was assumed that the knee angular velocity reflects indirectly the muscle shortening/lengthening velocity. However, the macroscopic “quasi-isometric,” “concentric,” and “eccentric” modes seen at the knee and hip articulations levels may not be representative of the contractile aspects at the muscle level (real fascicule length and shortening velocity) (Fukunaga et al., 1997; Hauraix et al., 2013). Indeed, even if the angular velocity of knee extension is kept constant, the shortening velocity of a fascicle is dependent on the force applied to the muscle-tendon complex (Ichinose et al., 2000). Additionally, the muscle-tendon interactions complicate the interpretation of simple movements (Ishikawa et al., 2005). For example, during walking, the joint indicated an eccentric contraction mode whereas the “*in vivo*” mode was isometric (Fukunaga et al., 2001). Nevertheless, we believe that this approach may be simple and useful for strength and conditioning coaches to implement specific exercises.

Previous studies have often zoomed on a restrained number of gates and runs, limiting the generalizability of their results to alpine ski racing. However, we selected a large number of run and cycles across trials and course designs to provide a true representation of the knee and hip joint kinematics and their variability in skiers. Importantly, the study used miniaturized wireless sensors to avoid any disturbance from real ski training (e.g., no backpack) and be representative of elite alpine ski racing.

It should be acknowledged that the filtering choice may have hindered differences between SL and the other disciplines due to an average underestimation of 8% in the signal amplitude. Indeed, edge's effects were unavoidable as the signal of interest was very close to the noise despite the very selective filter. However, despite the high constraints of the environment, the filter characteristics gave good results and represented an acceptable compromise of signal processing for this dataset (Figure 1).

It must be recognized that the intra-run (i.e., cycles) and inter-runs variability was taken into account by the factor “skier-session” for which a random effect was applied. However, the global variability of the skier-session (subject and course setting of the day) does not allow to separate the variability due to the skier alone, the day of testing alone (course setting, slope conditions), or the run/cycle number. As such, including the turn number within the analyses would provide an additional layer of information. However, this information would also be dependent of external (e.g., slope, snow conditions) and internal (e.g., fatigue) confounding factors. Therefore, as the aim of the current project was to provide a description of an “average” cycle, we did not want to separate the variability inherent to alpine ski racing (intra/inter runs) in this experiment.

Lastly, it should be acknowledged that some differences were statistically significant due to the very large amount of data but may not be functionally relevant due to their relatively small magnitude (~3–5°.s^−1^) with large SD. However, it should be mentioned that experienced skiers displayed a high degree of stability in their kinematic parameters despite the variability encountered on the slope in ski training as supported by a previous study showing a preserved posture in advanced skiers compared with beginners (Akutsu et al., 2008; Kiryu et al., 2011). Therefore, very small angle variations in the context of high-performance training may be meaningful.

### Perspectives

**Figure 6:**
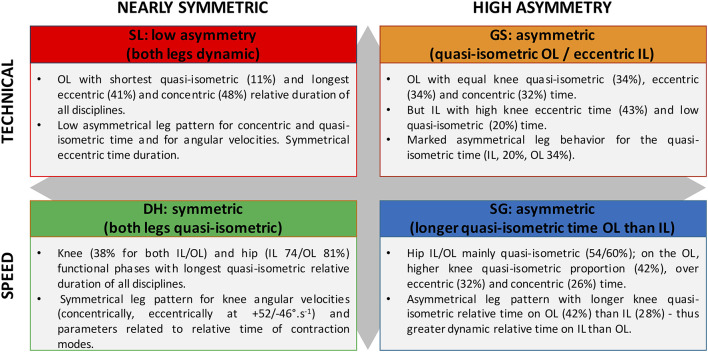
Synthetic overview of four alpine skiing disciplines based on joint kinematic characteristics (knee/hip angular velocity and position).

The present study characterized the knee and hip kinematic specificities of the alpine skiing Olympic disciplines. A synthetic overview is provided to help coaches to adapt their training to the features of each discipline (Figure 6). Especially, the quasi-isometric mode, evidenced as a key component of alpine skiing, which is known to generate training adaptations (Lum and Barbosa, 2019) that are joint angle-specific, especially through central (i.e., neural) pathways (Noorkõiv et al., 2014). Thus, it seems important to consider relative and absolute time as well as the specific angles at which the quasi-isometric mode occurs for each discipline during conditioning training.

Moreover, eccentric activity duration of the quadriceps muscle represents 30 to 43% on each leg, which is considered to be a unique feature of alpine skiing (Berg et al., 1995). Since eccentric muscle contractions require different activation strategies and programming processes by the central nervous system (Fang et al., 2004), regular application of eccentric exercise training is also warranted for alpine skiers. Of note, in alpine skiers, eccentric cycling training increased isometric torque only at greater quadriceps length probably due to sarcomerogenesis (Gross et al., 2010), As discipline-specific muscle adaptations depending on joint angle and contraction modes have been suggested in alpine skiers (Lešnik et al., 2012; Alhammoud et al., 2019), the present results may be used to adapt the training contents accordingly. Practically, the current results suggest that dryland strength and conditioning sessions should focus on slow movements at the specific angles of the OL and IL. It should however be acknowledged that the skiers are exposed to high frequency vibrations superimposed to the movement pattern (Figure 1), and that a loss of control may require a quick repositioning. As such, the slow velocity training should be completed with rapid force development (Jordan et al., 2015).

## Conclusion

This study examined the impact of skiing discipline and leg (outside/inside) on knee and hip angle-related parameters during real on-hill training. The continuous recording performed in this unique project in elite alpine skiers allowed us to characterize the presence of concentric, quasi-isometric and eccentric actions. The proportion of those phases significantly depended on the discipline in interaction with the inside/outside leg. The results showed a lower knee quasi-isometric duration on OL in SL than other disciplines, suggesting a highly dynamic style. Quasi-isometric mode was significantly longer on OL than IL in GS and SG but was significantly longer on IL than OL in SL. Thus, GS and SG showed similarities, with a significantly faster knee eccentric mean angular velocity on IL than OL whereas SL showed an opposite pattern. These considerations may be used to train the open and closed angles with specific contraction modes and exercises. Moreover, the “polyvalent” skiers practicing GS and SG may present some similarities in their muscular properties, challenging the traditional partition of the speed (DH/SG) and technical (SL/GS) disciplines.

## Data Availability Statement

The raw data supporting the conclusions of this article will be made available by the authors, upon reasonable request, to any qualified researcher.

## Ethics Statement

The studies involving human participants were reviewed and approved by Inter-University Laboratory of Human Movement Biology. The patients/participants provided their written informed consent to participate in this study.

## Author Contributions

MA, CHau, and BM designed the study. MA collected the data. MA, CHan, FM, CHau, and BM analyzed the data and drafted the manuscript. All authors revised and approved the manuscript.

## Conflict of Interest

The authors declare that the research was conducted in the absence of any commercial or financial relationships that could be construed as a potential conflict of interest.

## References

[B1] Akutsu T. Kiryu T. Ushiyama Y. Murayama T. (2008). Evaluation of functional activities during skiing exercise by knee joint angles and surface EMG signals. Trans. Soc. Instrum. Control Eng. 44, 905–910. 10.9746/ve.sicetr1965.44.905

[B2] Alhammoud M. Morel B. Hansen C. Wilson M. Mecca R. Nael E.. (2019). Discipline and sex differences in angle-specific isokinetic analysis in elite skiers. Int. J. Sports Med. 40, 317–330. 10.1055/a-0850-0016 30856671

[B3] Ball K. (2008). Biomechanical considerations of distance kicking in Australian rules football. Sports Biomech. 7, 10–23. 10.1080/14763140701683015 18341133

[B4] Berg H. E. Eiken O. (1999). Muscle control in elite alpine skiing. Med. Sci. Sports Exerc. 31, 1065–1067. 10416571 10.1097/00005768-199907000-00022

[B5] Berg H. E. Eiken O. Tesch P. A. (1995). Involvement of eccentric muscle actions in giant slalom racing. Med. Sci. Sports Exerc. 27, 1666–1670. 8614323

[B6] Brysbaert M. Stevens M. (2018). Power analysis and effect size in mixed effects models: a tutorial. J. Cogn. 1:9. 10.5334/joc.10 31517183 PMC6646942

[B7] Cnaan A. Laird N. M. Slasor P. (1997). Using the general linear mixed model to analyse unbalanced repeated measures and longitudinal data. Stat. Med. 16, 2349–2380. 10.1002/(sici)1097-0258(19971030)16:20<2349::aid-sim667>3.0.co;2-e 9351170

[B8] Connick M. J. Li F.-X. (2015). Prolonged cycling alters stride time variability and kinematics of a post-cycle transition run in triathletes. J. Electromyogr. Kinesiol. 25, 34–39. 10.1016/j.jelekin.2014.08.009 25282575

[B9] Falda-Buscaiot T. Hintzy F. Rougier P. Lacouture P. Coulmy N. (2017). Influence of slope steepness, foot position and turn phase on plantar pressure distribution during giant slalom alpine ski racing. PLoS ONE 12:e0176975. 10.1371/journal.pone.0176975 28472092 PMC5417654

[B10] Fang Y. Siemionow V. Sahgal V. Xiong F. Yue G. H. (2004). Distinct brain activation patterns for human maximal voluntary eccentric and concentric muscle actions. Brain Res. 1023, 200–212. 10.1016/j.brainres.2004.07.035 15374746

[B11] Fasel B. Spörri J. Gilgien M. Gerber N. Falbriard M. Müller E. (2016). IMU and GNSS-based turn switch detection in alpine ski racing, in Science and Skiing VII, eds Müller E. Kröll J. Lindinger S. Pfusterschmied J. Stöggl T. (Aachen: Meyer and Meyer Sport), 86–92.

[B12] Federolf P. von Tscharner V. Haeufle D. Nigg B. Gimpl M. Müller E. (2009). Vibration exposure in alpine skiing and consequences for muscle activation levels, in Science and Skiing IV, eds Müller E. Lindinger S. Stöggl T. (Maidenhead: Meyer and Meyer Verlag), 19–25.

[B13] Fukunaga T. Ichinose Y. Ito M. Kawakami Y. Fukashiro S. (1997). Determination of fascicle length and pennation in a contracting human muscle *in vivo*. J. Appl. Physiol. 82, 354–358. 10.1152/jappl.1997.82.1.354 9029238

[B14] Fukunaga T. Kubo K. Kawakami Y. Fukashiro S. Kanehisa H. Maganaris C. N. (2001). *In vivo* behaviour of human muscle tendon during walking. Proc. Biol. Sci. 268, 229–233. 10.1098/rspb.2000.1361 11217891 PMC1088596

[B15] Gilgien M. Kröll J. Spörri J. Crivelli P. Müller E. (2018a). Application of dGNSS in alpine ski racing: basis for evaluating physical demands and safety. Front. Physiol. 9:145. 10.3389/fphys.2018.00145 29559918 PMC5845727

[B16] Gilgien M. Reid R. Raschner C. Supej M. Holmberg H.-C. (2018b). The training of olympic alpine ski racers. Front. Physiol. 9:1772. 10.3389/fphys.2018.01772 30622477 PMC6308179

[B17] Gross M. Lüthy F. Kroell J. Müller E. Hoppeler H. Vogt M. (2010). Effects of eccentric cycle ergometry in alpine skiers. Int. J. Sports Med. 31, 572–576. 10.1055/s-0030-1254082 20464646

[B18] Hauraix H. Nordez A. Dorel S. (2013). Shortening behavior of the different components of muscle-tendon unit during isokinetic plantar flexions. J. Appl. Physiol. 115, 1015–1024. 10.1152/japplphysiol.00247.2013 23887903

[B19] Hawkins D. Hull M. L. (1990). A method for determining lower extremity muscle-tendon lengths during flexion/extension movements. J. Biomech. 23, 487–494. 10.1016/0021-9290(90)90304-l 2373721

[B20] Higbie E. J. Cureton K. J. Warren G. L. Prior B. M. (1996). Effects of concentric and eccentric training on muscle strength, cross-sectional area, and neural activation. J. Appl. Physiol. 81, 2173–2181. 10.1152/jappl.1996.81.5.2173 8941543

[B21] Hintermeister R. A. Lange G. W. O'Connor D. D. Dillman C. J. Steadman J. R. (1997). Muscle activity of the inside and outside leg in slalom and giant-slalom skiing, in Science and Skiing, eds Müller E. Schwameder H. Kornexl E. Raschner C. (London: Taylor and Francis), 141–149.

[B22] Hintermeister R. A. O'Connor D. D. Dillman C. J. Suplizio C. L. Lange G. W. Steadman J. R. (1995). Muscle activity in slalom and giant slalom skiing. Med. Sci. Sports Exerc. 27, 315–322. 7752856

[B23] Ichinose Y. Kawakami Y. Ito M. Kanehisa H. Fukunaga T. (2000). *In vivo* estimation of contraction velocity of human vastus lateralis muscle during “isokinetic” action. J. Appl. Physiol. 88, 851–856. 10.1152/jappl.2000.88.3.851 10710378

[B24] Ishikawa M. Komi P. V. Grey M. J. Lepola V. Bruggemann G.-P. (2005). Muscle-tendon interaction and elastic energy usage in human walking. J. Appl. Physiol. 99, 603–608. 10.1152/japplphysiol.00189.2005 15845776

[B25] Jordan M. J. Aagaard P. Herzog W. (2015). Rapid hamstrings/quadriceps strength in ACL-reconstructed elite alpine ski racers. Med. Sci. Sports Exerc. 47, 109–119. 10.1249/MSS.0000000000000375 24824771

[B26] Kiryu T. Murayama T. Ushiyama Y. (2011). Influence of muscular fatigue on skiing performance during parallel turns. Conf. Proc. IEEE Eng. Med. Biol. Soc. 2011, 8187–8190. 10.1109/IEMBS.2011.6092019 22256242

[B27] Kröll J. Schiefermüller C. Birklbauer J. Müller E. (2005). Inline-skating as a dry land modality for slalom racers-electromyographic and dynamic similarities and differences, in Science and Skiing III, eds. Mueller E. Lindinger S. Stoeggl T. (Maidenhead: Meyer and Meyer Sport), 76–87.

[B28] Kröll J. Spörri J. Fasel B. Müller E. Schwameder H. (2015a). Type of muscle control in elite alpine skiing – is it still the same than in 1995?, in Science and Skiing VI, eds Müller E. Kröll J. Lindinger S. Pfusterschmied J. Stöggl T. (Aachen: Meyer and Meyer Sport), 56–64.

[B29] Kröll J. Spörri J. Kandler C. Fasel B. Müller E. Schwameder H. (2015b). Kinetic and kinematic comparison of alpine ski racing disciplines as a base for specific conditioning regimes, in ISBS-Conference Proceedings Archive, eds Colloud F. Domalain M. Monnet T. (Poitiers), 33.

[B30] Kröll J. Wakeling J. M. Seifert J. G. Müller E. (2010). Quadriceps muscle function during recreational alpine skiing. Med. Sci. Sports Exerc. 42, 1545–1556. 10.1249/MSS.0b013e3181d299cf 20068490

[B31] Kuznetsova A. Brockhoff P. B. Christensen R. H. B. (2017). lmerTest package: tests in linear mixed effects models. J. Stat. Softw. 82, 1–26. 10.18637/jss.v082.i13

[B32] LeMaster R. (2010). Ultimate Skiing. Champaign, IL: Human Kinetics.

[B33] Lenth R. (2019). Data From: emmeans: Estimated Marginal Means aka Least-Squares Means. R package version 1.4. Available online at: https://CRAN.R-project.org/package=emmeans

[B34] Lešnik B. Šimuniþ B. Žvan M. Pišot R. (2012). Adaptation of vastii muscles in top skiers from different alpine skiing disciplines, in Science and Skiing V, eds Müller E. Lindinger S. Stöggl T. (Maidenhead: Meyer and Meyer Sport), 251–262.

[B35] Lum D. Barbosa T. M. (2019). Brief review: effects of isometric strength training on strength and dynamic performance. Int. J. Sports Med. 40, 363–375. 10.1055/a-0863-4539 30943568

[B36] Manning W. G. Mullahy J. (2001). Estimating log models: to transform or not to transform? J. Health Econ. 20, 461–494. 10.1016/s0167-6296(01)00086-8 11469231

[B37] Meyer F. Prenleloup A. Schorderet A. (2019). Development of a new embedded dynamometer for the measurement of forces and torques at the ski-binding interface. Sensors 19:4324. 10.3390/s19194324 31591295 PMC6806195

[B38] Minetti A. (2016). Concentric, isometric and eccentric contractions: which dominates alpine skiing?, in Science and Skiing VII, eds Müller E. Kröll J. Lindinger S. Pfusterschmied J. Spörri J. Stöggl T. (Maidenhead: Meyer and Meyer Sport), 23–29.

[B39] Moon J. Koo D. Kim K. Shin I. Kim H. Kim J. (2015). Effect of ski simulator training on kinematic and muscle activation of the lower extremities. J. Phys. Ther. Sci. 27, 2629–2632. 10.1589/jpts.27.2629 26357449 PMC4563330

[B40] Morel B. Lapole T. Liotard C. Hautier C. (2019). Critical peripheral fatigue thresholds among different force-velocity conditions: an individual-based model approach. Front. Physiol. 10:875. 10.3389/fphys.2019.00875 31379595 PMC6646582

[B41] Müller E. Benko U. Raschner C. Schwameder H. (2000). Specific fitness training and testing in competitive sports. Med. Sci. Sports Exerc. 32, 216–220. 10.1097/00005768-200001000-00032 10647552

[B42] Nemec B. Kugovnik O. Supej M. (2001). Influence of the ski side cut on vibrations in alpine skiing, in Science and Skiing II, eds Müller E. Schwameder H. Raschner C. Lindinger S. Kornexl E. (Hamburg: Verlag Dr. Kovac), 232–241.

[B43] Nilsson J. Haugen P. (2004). Knee angular displacement and extensor muscle activity in telemark skiing and in ski-specific strength exercises. J. Sports Sci. 22, 357–364. 10.1080/02640410310001641557 15161109

[B44] Noorkõiv M. Nosaka K. Blazevich A. J. (2014). Neuromuscular adaptations associated with knee joint angle-specific force change. Med. Sci. Sports Exerc. 46, 1525–1537. 10.1249/MSS.0000000000000269 24504427

[B45] Padulo J. Di Capua R. Viggiano D. (2012). Pedaling time variability is increased in dropped riding position. Eur. J. Appl. Physiol. 112, 3161–3165. 10.1007/s00421-011-2282-8 22183087

[B46] Panizzolo F. A. Marcolin G. Petrone N. (2013). Comparative evaluation of two skiing simulators as functional training devices for recreational skiers. J. Sports Sci. Med. 12, 151–158. 24149739 PMC3761755

[B47] Pozzo R. Canclini A. Baroni G. Benedetti D. D'Ottavio S. (2010). 3-D kinematic and kinetics analysis of slalom in elite skiers at the Bormio World Cup ski finals in 2008, in Science and Skiing V, eds Müller E. Lindinger S. Stöggl T. (Maidenhead: Meyer and Meyer Sport), 355–363.

[B48] Raschner C. Schiefermüller C. Zallinger G. Hofer E. Brunner F. (2001). Carving turns versus traditional parallel turns–a comparative biomechanical analysis, in Science and Skiing II, eds Müller E. Schwameder H. Raschner C. Lindinger S. Kornexl E. (Hamburg: Verlag Dr. Kovac), 203–217.

[B49] Rohatgi A. (2015). Data From: WebPlotDigitalizer: HTML5 Based Online Tool to Extract Numerical Data From Plot Images. Available online at: https://automeris.io/WebPlotDigitizer

[B50] Stöggl T. Kröll J. Helmberger R. Cudrigh M. Müller E. (2018). Acute effects of an ergometer-based dryland alpine skiing specific high intensity interval training. Front. Physiol. 9:1485. 10.3389/fphys.2018.01485 30405439 PMC6200917

[B51] Struzik A. Konieczny G. Stawarz M. Grzesik K. Winiarski S. Rokita A. (2016). Relationship between lower limb angular kinematic variables and the effectiveness of sprinting during the acceleration phase. Appl. Bionics Biomech. 2016:7480709. 10.1155/2016/7480709 27516724 PMC4969523

[B52] Supej M. Holmberg H.-C. (2019). Recent kinematic and kinetic advances in olympic alpine skiing: pyeongchang and beyond. Front. Physiol. 10:111. 10.3389/fphys.2019.00111 30842740 PMC6391578

[B53] Supej M. Kugovnik O. Nemec B. (2003). Kinematic determination of the beginning of a ski turn. Kinesiol. Slov. 9, 5–11.

[B54] Supej M. Saetran L. Oggiano L. Ettema G. Šarabon N. Nemec B. (2013). Aerodynamic drag is not the major determinant of performance during giant slalom skiing at the elite level. Scand. J. Med. Sci. Sports 23, e38–e47. 10.1111/sms.12007 23121340

[B55] Turnbull J. R. Kilding A. E. Keogh J. W. L. (2009). Physiology of alpine skiing. Scand. J. Med. Sci. Sports 19, 146–155. 10.1111/j.1600-0838.2009.00901.x 19335589

[B56] Vogt M. Hoppeler H. H. (2014). Eccentric exercise: mechanisms and effects when used as training regime or training adjunct. J. Appl. Physiol. 1985 116, 1446–1454. 10.1152/japplphysiol.00146.2013 24505103

[B57] Westfall J. Kenny D. A. Judd C. M. (2014). Statistical power and optimal design in experiments in which samples of participants respond to samples of stimuli. J. Exp. Psychol. Gen. 143, 2020–2045. 10.1037/xge0000014 25111580

[B58] Zeglinksi C. M. Swanson S. C. Self B. P. Greenwald R. M. (1998). Muscle activity in the slalom turn of alpine skiing and in-line skating. Int. J. Sports Med. 19, 447–454. 10.1055/s-2007-971943 9839840

